# GSAMDA: a computational model for predicting potential microbe–drug associations based on graph attention network and sparse autoencoder

**DOI:** 10.1186/s12859-022-05053-7

**Published:** 2022-11-18

**Authors:** Yaqin Tan, Juan Zou, Linai Kuang, Xiangyi Wang, Bin Zeng, Zhen Zhang, Lei Wang

**Affiliations:** 1 Key Laboratory of Hunan Province for Internet of Things and Information Security, Xiangtan University, Xiangtan, 411105 China; 2 Institute of Bioinformatics Complex Network Big Data, Changsha University, Changsha, 410022 China; 3 Big Data Innovation and Entrepreneurship Education Center of Hunan Province, Changsha University, Changsha, 410022 China

**Keywords:** Microbe–drug associations, Graph attention network-based autoencoder, Sparse autoencoder

## Abstract

**Background:**

Clinical studies show that microorganisms are closely related to human health, and the discovery of potential associations between microbes and drugs will facilitate drug research and development. However, at present, few computational methods for predicting microbe–drug associations have been proposed.

**Results:**

In this work, we proposed a novel computational model named GSAMDA based on the graph attention network and sparse autoencoder to infer latent microbe–drug associations. In GSAMDA, we first built a heterogeneous network through integrating known microbe–drug associations, microbe similarities and drug similarities. And then, we adopted a GAT-based autoencoder and a sparse autoencoder module respectively to learn topological representations and attribute representations for nodes in the newly constructed heterogeneous network. Finally, based on these two kinds of node representations, we constructed two kinds of feature matrices for microbes and drugs separately, and then, utilized them to calculate possible association scores for microbe–drug pairs.

**Conclusion:**

A novel computational model is proposed for predicting potential microbe–drug associations based on graph attention network and sparse autoencoder. Compared with other five state-of-the-art competitive methods, the experimental results illustrated that our model can achieve better performance. Moreover, case studies on two categories of representative drugs and microbes further demonstrated the effectiveness of our model as well.

## Background

Microorganisms, including bacteria, viruses, archaea, fungi and protozoa, are dynamic, diverse and complex genetic reservoirs that exist in interactive flux, colonize human cells, and play significant roles in human beings [1]. The microbial function is to protect the pathogens, improve and enhance metabolism and immunity capability [2]. For example, microbes can resist the invasion of opportunistic pathogens [3], promote the synthesis of sugar metabolism and synthesis the necessary vitamins to boost T-cell responses [4]. Maintaining the homeostasis of internal environment of organisms is inseparable from the regulation of microorganisms [5]. Unusual growth or decline of microorganisms will influence human health and cause diseases, such as obesity [6], inflammatory bowel disease [7], and even cancer [8]. For instance, pathogens, including bacteria and viruses, may cause infectious diseases such as the COVID-19 [9]. Also, while using drugs to treat microbe-caused diseases, the microbiome may affect the physiological action of drugs in turn. Several studies have shown that not only microbial metabolism can significantly affect the clinical response to drugs, but also the administration of drugs can similarly affect the microbiome [1, 10, 11]. Hence, uncovering potential associations between microbes and drugs will be helpful for the development of drugs and the treatment of human diseases. Due to the high cost and time-consuming of clinical and biological experiments, it is obvious that effective computational approaches for predicting possible microbe–drug associations will be useful complements of traditional web-lab experiments.

Recently, researchers have published multiple databases such as MDAD [12] and aBiofilm [13], which include a large number of experimentally validated microbe–drug associations. And based on these databases, a series of calculation methods have been proposed to detect latent microbe–drug associations and achieved a certain degree of effects. For instance, Zhu et al. proposed a method called HMDAKATZ by adopting KATZ metric to infer potential microbe–drug associations [14]. Long et al. designed a calculation framework named HNERMDA for possible microbe–drug association prediction through combining metapath2vec with bipartite network recommendation [15]. Furthermore, a computational model called LRLSMDA was proposed in reference [16] for identifying microbe–drug associations based on the Laplacian Regularized Least Square algorithm. Literature [17] introduced a calculation scheme named GCNMDA based on the Graph Convolutional Network (GCN) and Conditional Random Field (CRF) to discover associations between microbes and drugs. In reference [18], a method called EGATMDA was designed based on the framework of graph attention networks to predict possible microbe–drug associations. Additionally, Deng et al. conceived a calculation model named Graph2MDA through applying a variational graph autoencoder to infer microbe–drug associations [19].

Most of the above methods took multiple node features into account and fed them into the same model for prediction. Hence, considering that different node features can be learned by different models may have better performance, we classified node features as topological features and attribute features and learn the representations of these two features through graph attention network(GAT) and sparse autoencoder(SAE) respectively. GAT can propagate the information from local neighbors to learn effective representations and has been widely and successfully used in the field of association prediction such as Long et al. [18], Liu et al. [20]. SAE can extract relatively sparse and useful features by introducing a sparse penalty term on autoencoder [21].

In this paper, we introduced a novel calculation method called GSAMDA based on the graph attention network (GAT) and the sparse autoencoder (SAE) to predict potential microbe–drug associations. In GSAMDA, a heterogeneous network would be constructed first based on the Gaussian interaction profile (GIP) kernel similarity and Hamming interaction profile (HIP) similarity for microbes and drugs. And then, for each node in the heterogeneous network, a unique topological representation would be learned by adopting a GAT-based autoencoder. Simultaneously, based on multiple features of microbes and drugs, we would further apply SAE to learn a unique attribute representation for each node in the heterogeneous network as well. Thereafter, through combining these two types of node representations with multiple features of microbes and drugs, such as drug structure similarity, microbe functional similarity, drug–disease associations and microbe–disease associations, a unique feature matrix would be built for each node in the heterogeneous network, which would be utilized to obtain predicted scores for possible microbe–drug associations. Finally, in order to verify the prediction performance of GSAMDA, we performed case studies and intensive comparison experiments based on two well-known public databases, and results demonstrated that GSAMDA outperformed five state-of-the-art competitive methods, which means that GSAMDA not only can achieve satisfactory predictive performance, but also may be a kind of useful tool for potential microbe–drug association prediction in the future.

## Materials and methods

### Data sources

In this manuscript, we first downloaded known microbe–drug associations from two public databases such as MDAD (http://www.chengroup.cumt.edu.cn/MDAD/) and aBiofilm (http://bioinfo.imtech.res.in/manojk/abiofilm/) separately. As a result, we obtained 2470 clinically or experimentally verified microbe–drug associations between 1373 drugs and 173 microbes from the MDAD, while 2884 known microbe–drug associations between 1720 drugs and 140 microbes from the aBiofilm. And then, we further collected known drug–disease associations and known microbe–disease associations from the dataset proposed by Wang et al. [22] as well. During the experiment, only diseases associating with at least one drug and one microbe in the MDAD or aBiofilm, and associations related with these diseases, would be kept. Hence, we finally obtained 109 different diseases, 232 different drugs, 1121 different drug–disease associations and 402 different microbe–disease associations from the MDAD, and 72 different diseases, 103 different drugs, 435 different drug–disease associations and 254 different microbe–disease associations from the aBiofilm. The detailed numbers of these aforementioned data were shown in the following Table 1.

**Table 1:** The detailed numbers of microbes, drugs, diseases and related associations in the MDAD and aBiofilm

Type (MDAD/aBiofilm)	Microbes	Drugs	Diseases	associations
Microbe–drug associations	173/140	1373/1720	–	2470/2884
Microbe–disease associations	73/59	–	109/72	402/254
Drug–disease associations	–	233/103	109/72	1121/435

### Methods

**Fig. 1:**
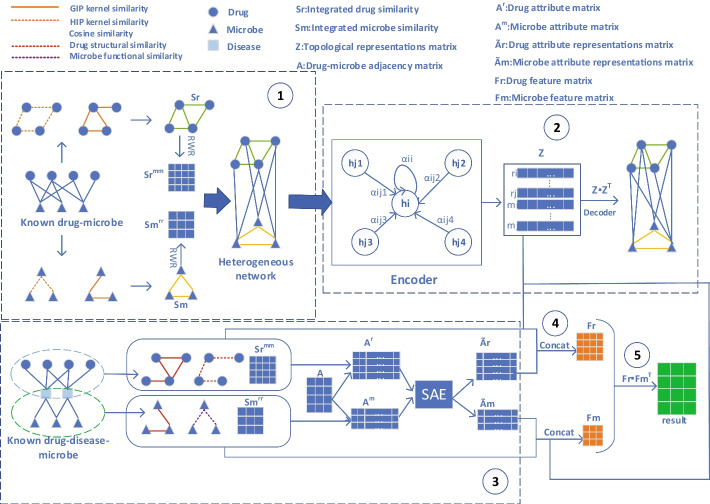
The overall architecture of GSAMDA

As shown in Fig. 1, GSAMDA mainly consists of five parts:

Step 1. Constructing the heterogeneous network *HN* by adopting integrated microbe similarities and drug similarities;

Step 2. Learning topological representations for nodes in *HN* based on the GAT;

Step 3. Learning attribute representations for nodes in *HN* based on the SAE;

Step 4. Constructing feature matrices for nodes in *HN* through combining their topological representations and attribute representations with multiple original attributes of them;

Step 5. Computing possible association scores for microbe–drug pairs based on their feature matrices.

### Constructing the heterogeneous network *HN*

In this section, based on newly downloaded drugs, microbes and known microbe–drug associations, we would build the heterogeneous network *HN* as follows.

Firstly, we defined *A*∈${R}^{{n}_{r}\times {n}_{m}}$ as an adjacency matrix, where *n*_*r*_ and *n*_*m*_ denote the numbers of newly downloaded drugs and microbes separately. In *A*, for any given drug *r*_*i*_ and microbe *m*_*j*_, if there is a known association between them, then there is *A*_*ij*_ = 1, otherwise there is *A*_*ij*_ = 0.

Secondly, let *A*(*r*_*i*_) and *A*(*m*_*j*_) denote the *i*-th row and the *j*-th column of *A* respectively, then for any two given drugs *r*_*i*_ and *r*_*j*_, we would estimate the GIP kernel similarity ${S}_{r}^{GIP}({r}_{i},{r}_{j})\in {R}^{{n}_{r}\times {n}_{r}}$ between them as follows:

1 $${S}_{r}^{GIP}({r}_{i},{r}_{j})=exp(-{\gamma }_{r}{||A({r}_{i})-A({r}_{j})||}^{2})$$

2 $${\gamma }_{r}=1/\left(\frac{1}{{n}_{r}}\sum_{i=1}^{{n}_{r}}{||A({r}_{i})||}^{2}\right)$$

Similarly, for any two given microbes *m*_*i*_ and *m*_*j*_, we would evaluate the GIP kernel similarity ${S}_{m}^{GIP}({m}_{i},{m}_{j})\in {R}^{{n}_{m}\times {n}_{m}}$ between them as follows:

3 $${S}_{m}^{GIP}({m}_{i},{m}_{j})=exp(-{\gamma }_{m}{||A({m}_{i})-A({m}_{j})||}^{2})$$

4 $${\gamma }_{m}=1/\left(\frac{1}{{n}_{m}}\sum_{i=1}^{{n}_{m}}{||A({m}_{i})||}^{2}\right)$$

Here, ||·|| is the Frobenius norm.

Thirdly, inspired by the work proposed by Xu et al. [23], we further adopted the HIP similarity to measure the similarities between drugs or microbes based on the assumption that two nodes will have lower similarity when their interaction profiles are more different. To be specific, for any two given drugs *r*_*i*_ and *r*_*j*_, the HIP similarity ${S}_{r}^{HIP}({r}_{i},{r}_{j})\in {R}^{{n}_{r}\times {n}_{r}}$ between them would be computed as follows:

5 $${S}_{r}^{HIP}({r}_{i},{r}_{j})=1-\frac{|A({r}_{i})!=A({r}_{j})|}{|A({r}_{i})|}$$

where $|A({r}_{i})!=A({r}_{j})|$ denotes the number of different elements between the profiles *A* $({r}_{i})$ and $A({r}_{j})$, and $|A({r}_{i})|$ represents the number of elements in $A({r}_{i})$. Similarly, for any two given microbes *m*_*i*_ and *m*_*j*_, the HIP similarity ${S}_{m}^{HIP}({m}_{i},{m}_{j})\in {R}^{{n}_{m}\times {n}_{m}}$ between them could be estimated as follows:

6 $${S}_{m}^{HIP}({m}_{i},{m}_{j})=1-\frac{|A({m}_{i})!=A({m}_{j})|}{|A({m}_{i})|}$$

Finally, considering that the values in both the matrices ${S}_{r}^{GIP}$ and ${S}_{r}^{HIP}$ range from 0 to 1, we could combine these two matrices into a new matrix ${S}_{r}\in {R}^{{n}_{r}\times {n}_{r}}$ as follows:

7 $${S}_{r}=({S}_{r}^{GIP}+{S}_{r}^{HIP})/2$$

Similarly, a novel matrix ${S}_{m}\in {R}^{{n}_{m}\times {n}_{m}}$ could be obtained by integrating ${S}_{m}^{GIP}$ and ${S}_{m}^{HIP}$ as follows:

8 $${S}_{m}=({S}_{m}^{GIP}+{S}_{m}^{HIP})/2$$

Thereafter, a matrix $N\in {R}^{{{(n}_{r}+n}_{m})\times {{(n}_{r}+n}_{m})}$ could be constructed through combining ${S}_{r}$ and ${S}_{m}$ with the adjacency matrix *A* as follows:

9 $$N=\left[\begin{array}{cc}{S}_{r}& A\\ {A}^{T}& {S}_{m}\end{array}\right]$$

Here, ${A}^{T}$ is the transposed matrix of *A*.

Obviously, based on above matrix *N*, we can easily design a heterogeneous network *HN* consisting of ${{n}_{r}+n}_{m}$ different nodes, in which, there is an edge between any two nodes *i* and *j*, if and only if there is $N(i,j)\ne 0$.

### Learning topological representations for nodes in *HN*

The graph attention network (GAT) is an extension of the graph convolution network, it can overcome some shortcomings of graph convolution by using the masked self-attentional layers, which allows implicitly different weights to be assigned to different nodes in an adjacent set of nodes [24]. In this section, we would construct a GAT and take the network *HN* as its input to learn topological representations for nodes in *N* according to the following steps:

**Step1 (Encoder)**: For any given node *i* in *HN*, let ${N}_{i}$ denote the set of neighboring nodes of *i* in *N*, then, for any node $j\in {N}_{i}$, the GAT would first calculate the attention score ${\alpha }_{ij}$ between *i* and *j* according to the following formulae:

10 $${\alpha }_{ij}=softmax\left({e}_{ij}\right)=\frac{\mathrm{exp}({e}_{ij})}{{\sum }_{k\in {N}_{i}}\mathrm{exp}({e}_{ik})}$$

11 $$softmax(x)=\frac{1}{1+{e}^{-x}}$$

12 $${e}_{ij}=LeakyRelu(\alpha [W{h}_{i}||W{h}_{j}])$$

13 $$LeakyRelu(x)=\left\{\begin{array}{c}x x>0\\ \mu x otherwise\end{array}\right.$$

Here, $\alpha$ represents the computational operation of self-attention, *W* is the matrix of trainable weights, *h*_*i*_ denotes the feature representation of the node *i* (i.e., the *i-th* row of *N*), $\mu$ is the hypermeter and || denotes the concatenation operation.

Subsequently, the GAT would multiply the attention score ${\alpha }_{ij}$ with the feature representation ${h}_{j}$ of each node in ${N}_{i}$ and sum all these products up as follows:

14 $${h}_{i}=\sigma \left(\sum_{j\in {N}_{i}}{\alpha }_{ij}W{h}_{j}\right)$$

Here, $\sigma$ denotes the activation function.

After above Encoder step, obviously, we could obtain a matrix $$Z=\left[\begin{array}{c}{Z}^{r}\\ {Z}^{m}\end{array}\right]\in {R}^{({n}_{r}+{n}_{m})*l}$$, where ${Z}^{r}$ and ${Z}^{m}$ represent the low-dimensional topological representation of drug nodes and microbe nodes in *HN* respectively.

**Step2(Decoder)** Based on the matrix $Z$, it was easy to see that we could take its inner product as a decoder:

15 $$ZZ=sigmoid(Z\bullet {Z}^{T})$$

16 $$sigmoid(x)=\frac{1}{1+{e}^{-x}}$$

**Step3(Optimization)** Considering that the decoded result *ZZ* should be close to the original inputted matrix *N*, we adopted the MSE loss function to compute the mean of the sum of squares of the differences between *ZZ* and *N* as follows:

17 $${L}_{MSE}=\frac{1}{{n}_{r}+{n}_{m}}\sum_{k=1}^{{n}_{r}+{n}_{m}}{||ZZ(k)-N(k)||}^{2}$$

where *ZZ*(*k*) and *N*(*k*) denote the *k*-th row of *ZZ* and *N* respectively.

Thereafter, based on the Eq. (16), we would adopt the Adam optimizer [25] to optimize the results of topological representations for nodes in *HN*.

### Learning attribute representations for nodes in *HN*

In this section, in order to effectively capture local and global topological intrinsic characteristics of nodes in *HN*, we further implemented an improved random walk with restart (RWR) on ${S}_{r}$, where the RWR was defined as follows [26]:

18 $${q}_{i}^{l+1}=\varphi M{q}_{i}^{l}+(1-\varphi ){\varepsilon }_{i}$$

Here, $\mathrm{\varphi }$ is the restart probability and set as 0.1, *M* denotes the transition probability matrix, and ${\varepsilon }_{i}$∈${R}^{1\times m}$ is the initial probability vector of the node *i*, which is defined as follows:

19 $${\varepsilon }_{ij}=\left\{\begin{array}{c}1 if i=j\\ 0 otherwise\end{array}\right.$$

Based on above RWR process, it was easy to know that we could obtain a novel matrix ${S}_{r}^{mm}$.

In addition, let ${n}_{d}$ denote the number of newly downloaded diseases, similar to construction of the adjacency matrix *A*, we could obtain an adjacency matrix *D*∈${R}^{{n}_{r}\times {n}_{d}}$ based on these newly-downloaded known drug–disease associations as well. And then, for any two given drug nodes *i* and *j* in *HN*, we could calculate the cosine similarity ${S}_{r}^{dis}(i,j)$ between them as follows:

20 $${S}_{r}^{dis}(i,j)=cos(D(i),D(j))=\frac{D(i)\cdot D(j)}{||D(i)||\times ||D(j)||}$$

Here, *D*(*i*) denotes the *i-th* row of *D*.

Moreover, in a similar way, we could further calculate the drug structural similarity matrix ${S}_{r}^{che}$ based on the dataset downloaded from the SIMCOMP2 [27], which measured the drug similarity based on the drug chemical structure information.

Hence, through integrating all these matrices *A*, ${S}_{r}^{mm}$, ${S}_{r}^{dis}$ and ${S}_{r}^{che}$, it is easy to see that we could obtain a novel drug attribute matrix ${A}^{r}$ as follows:

21 $${A}^{r}=[A;{S}_{r}^{che};{S}_{r}^{mm};{S}_{r}^{dis}]$$

Similarly, after applying the improved RWR on ${S}_{m}$, we could obtain a new matrix ${S}_{m}^{rr}$.

And in addition, through adopting the method proposed by Kamneva [28], which calculated the functional similarity between microbes based on a microbial protein–protein functional association network, we could obtain a new matrix ${S}_{m}^{f}$ as well. Here, in the microbial protein–protein functional association network, the nodes represent any gene family encoded by the genome and the edges denote genetic neighbor scores based on STRING database. The functional similarity between microbes was calculated as the ratio of the link scores connecting the two microbes to the sum of all the link scores of the two microbial gene families.

Moreover, similar to the construction of ${S}_{r}^{dis}$, based on the dataset of newly downloaded known microbe–disease associations, for any two given microbe nodes *i* and *j* in *HN*, we could calculate the cosine similarity ${S}_{m}^{dis}(i,j)$ between them in a similar way as well.

Hence, through integrating all these matrices ${A}^{T}$, ${S}_{m}^{f}$, ${S}_{m}^{rr}$ and ${S}_{m}^{dis}$, it is obvious that we could obtain a novel microbe attribute matrix ${A}^{m}$ as follows:

22 $${A}^{m}=[{A}^{T};{S}_{m}^{f};{S}_{m}^{rr};{S}_{m}^{dis}]$$

**Fig. 2:**
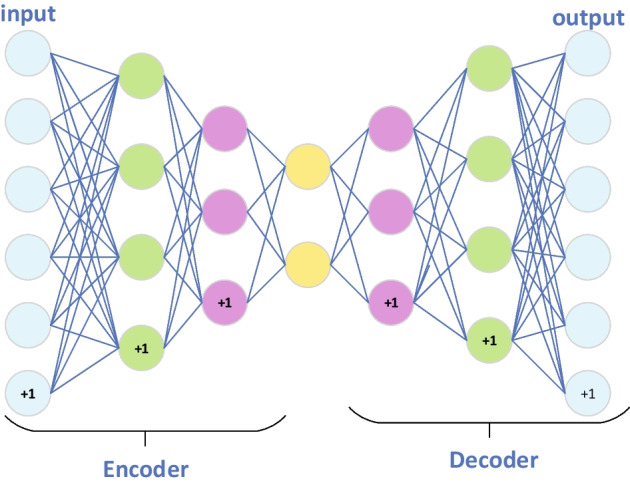
The overall architecture of GSAMDA

Thereafter, after taking above two kinds of attribute matrices ${A}^{r}$ and ${A}^{m}$ as input of the SAE respectively, we could learn a unique attribute representation for each node in *HN* as well, where the structure of SAE was shown in the following Fig. 2.

From observing above Fig. 2, it is easy to see that the SAE consists of the following steps:

**Step1(Encoder)** Let *h* and *x* represent the hidden layer and the input layer of the SAE respectively, the encoder could be formulated as follows:

23 $${h}_{W,b}=\sigma ({W}_{encoder}x(i)+{b}_{encoder})$$

**Step2(Decoder)** The decoder adopted the same structure as the encoder, which was defined as follows:

24 $${y}_{W,b}=\sigma ({W}_{decoder}h+{b}_{decoder})$$

where *W* is the weight matrix between two layers and *b* is the bias term.

Moreover, in order to ensure the sparsity of the hidden layer, we would add a penalty term in the SAE as follows:

25 $${P}_{penalty}=\sum_{t=1}^{{S}_{2}}KL(\rho ||\widehat{{\rho }_{t}})$$

where ${S}_{2}$ is the number of neurons in the hidden layer, $\widehat{{\rho }_{t}}$ represents the average activity of hidden neuron t, $KL(\rho ||\widehat{{\rho }_{t}})$ is the relative entropy between two Bernoulli random variables with mean $\rho$ and mean $\widehat{{\rho }_{t}}$ and is defined as follows:

26 $$KL(\rho ||\widehat{{\rho }_{t}})=\rho log\frac{\rho }{\widehat{{\rho }_{t}}}+(1-\rho )log\frac{1-\rho }{1-\widehat{{\rho }_{t}}}$$

Hence, after inputting the drug attribute matrix ${A}^{r}$ and the microbe attribute matrix ${A}^{m}$ into the SAE, we could obtain the output matrices ${A}^{rr}$ and ${A}^{mm}$ respectively.

**Step3(Optimization)** In the SAE, we adopted the MSE loss function and the Adam optimizer for optimization as well. During optimization, the sparse penalty term would be added to the loss function as follows (Taking the drug attribute matrix as an example):

27 $${L}_{sparse}=\frac{1}{{n}_{r}}\sum_{k=1}^{{n}_{r}}{||{A}^{rr}(k)-{A}^{r}(k)||}^{2}+\beta {P}_{penalty}$$

Here, $\beta$ is the weight of the sparse penalty and will be set to 0.1. ${A}^{rr}(k)$ and ${A}^{r}(k$) represent the *k-th* row of ${A}^{rr}$ and ${A}^{r}$ respectively.

After training the SAE, we could adopt the trained SAE to learn and obtain the low dimensional drug attribute representation matrix $\widetilde{{A}^{r}}\in {R}^{{n}_{r}*k}$ and microbe attribute representation matrix $\widetilde{{A}^{m}}\in {R}^{{n}_{m}*k}$ simultaneously.

### Constructing feature matrices for microbes and drugs

Based on above drug topological representation matrix ${Z}^{r}$, drug attribute representation matrix $\widetilde{{A}^{r}}$, drug structural similarity matrix ${S}_{r}^{che}$, drug cosine similarity matrix ${S}_{r}^{dis}$, drug similarity matrix ${S}_{r}^{mm}$ and the original adjacency matrix *A*, inspired by Xuan et al. [29], we could construct a novel drug feature matrix ${F}_{r}$ as follows:

28 $${F}_{r}=[{Z}^{r};\widetilde{{A}^{r}};{S}_{r}^{che};A;{S}_{r}^{dis};A;{S}_{r}^{mm};A]$$

Similarly, based on above microbe topological representation matrix ${Z}^{m}$, microbe attribute representation matrix $\widetilde{{A}^{m}}$, microbe functional similarity matrix ${S}_{m}^{f}$, microbe cosine similarity matrix ${S}_{m}^{dis}$, microbe similarity matrix ${S}_{m}^{rr}$ and the original adjacency matrix ${A}^{T}$, we can construct a novel microbe feature matrix ${F}_{m}$ as follows:

29 $${F}_{m}=[{Z}^{m};\widetilde{{A}^{m}};{A}^{T};{S}_{m}^{fun};{A}^{T}{;S}_{m}^{dis};{A}^{T};{S}_{m}^{rr}]$$

### Computing predicted scores for microbe–drug pairs

The multiplication of two vectors is an effective means of simulating the interaction, which emphasizes the commonality of the interaction and dilutes the difference information of the interaction. Hence, for any given drug ${r}_{i}$ and microbe ${m}_{j}$, we could obtain the predicted score between them by calculating the inner product of their feature representations as follows:

30 $$S({r}_{i},{m}_{j})=Sigmoid({F}_{r}({r}_{i})\bullet {{F}_{m}({m}_{j})}^{T})$$

Here, ${F}_{r}({r}_{i})$ denotes the *i-*th row of ${F}_{r}$ and ${F}_{m}({m}_{j})$ denotes the *j-*th row of ${F}_{m}$.

## Results

In this section, we first compared GSAMDA with five state-of-the-art competitive predictive methods based on databases MDAD and aBiofilm separately. And then, we conducted the hyperparameter sensitivity analysis to decide the best parameters. Finally, we implemented case studies on two selected drugs and two selected microbes to further demonstrate the performance of GSAMDA.

### Comparison with state-of-the-art methods

As predicting microbe–drug associations is a new problem, there are few computational methods and codes available, therefore, we would compare our method GSAMDA with some representative methods for link prediction problems in this section. Among them, HMDAKATZ [14] is a KATZ-based method proposed for microbe–drug associations prediction. LAGCN [30] is a graph convolutional network with attention mechanism based method designed to infer potential drug–disease associations. NTSHMDA [31] is a model based on random walk with restart for microbe–disease associations prediction. HMDA-Pred [32] integrated multiple data types and adopted the Network Consistency Projection (NCP) technique to detect latent microbe–disease associations. BPNNHMDA [33] designed a novel neural network to infer microbe–disease associations.

**Fig. 3:**
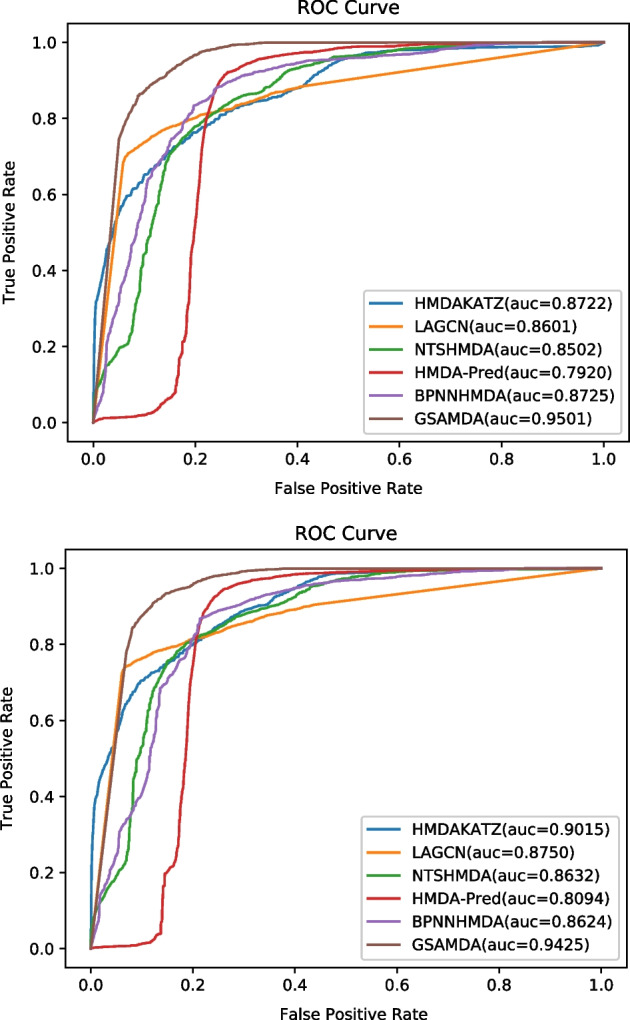
**a** ROC curves of six competitive methods based on the MDAD dataset. **b** ROC curves of six competitive methods based on the aBiofilm dataset

**Fig. 4:**
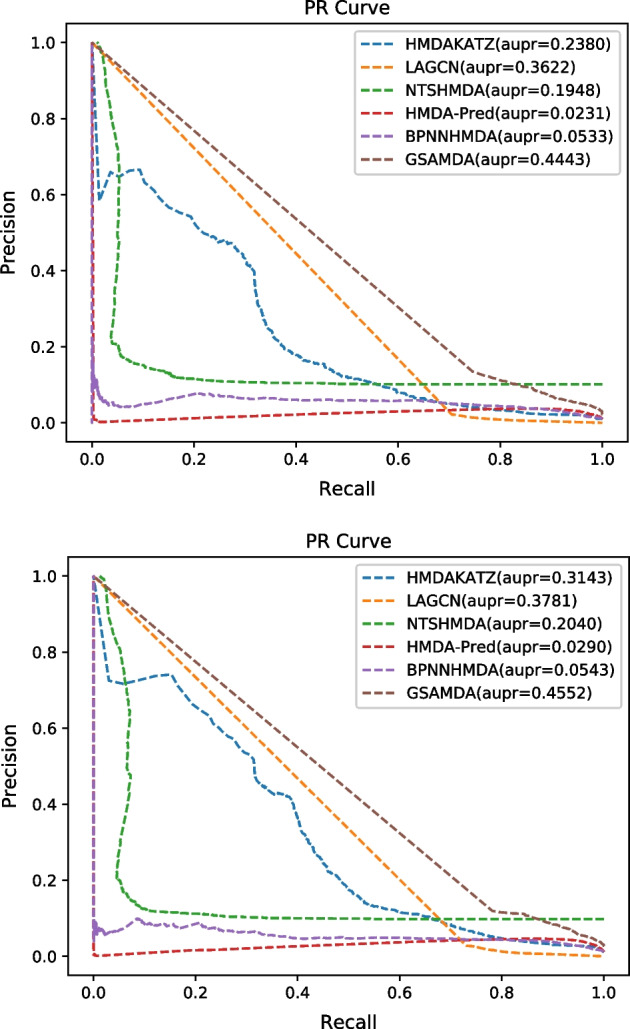
**a** PR curves of six competitive methods based on the MDAD dataset. **b** PR curves of six competitive methods based on the aBiofilm dataset

During experiments, we settled with the original parameters for all these competitive methods and ran them on the MDAD and aBiofilm datasets respectively for a fair comparison. In addition, we adopted the framework of fivefold cross validation (CV) in Cai et al. [34] to evaluate these methods, in which, we randomly selected 20% of known associations and 20% of unknown associations as the testing set, and the remaining 80% of known associations and unknown associations as the training set. We run the fivefold CV for 10 times and the AUROCs, AUPR and the best Accuracy of all compared methods were shown in Table 2. The best ROC curves and PR curves of these six competitive methods based on datasets MDAD and aBiofilm were drawn in Figs. 3 and 4, respectively.

**Table 2:** The AUCs, AUPRs and Accuracy of compared methods based on datasets MDAD and aBiofilm under fivefold CV

Methods	AUC		AUPR		Accuracy	
	MDAD	aBiofilm	MDAD	aBiofilm	MDAD	aBiofilm
HMDAKATZ	0.8712 ± 0.0010	0.8993 ± 0.0021	0.2327 ± 0.0068	0.3066 ± 0.0077	0.9774	0.9796
LAGCN	0.8533 ± 0.0070	0.8641 ± 0.0109	0.3571 ± 0.0051	0.3671 ± 0.0055	0.9413	0.9373
NTSHMDA	0.8483 ± 0.0020	0.8610 ± 0.0022	0.1892 ± 0.0056	0.1962 ± 0.0078	0.9896	**0.9882**
HMDA-Pred	0.7987 ± 0.0030	0.8053 ± 0.0040	0.0236 ± 0.0009	0.0284 ± 0.0006	0.9794	0.9806
BPNNHMDA	0.8410 ± 0.0320	0.8438 ± 0.0186	0.0319 ± 0.0105	0.0476 ± 0.0067	0.9894	0.9869
GSAMDA	**0.9496 ± 0.0005**	**0.9308 ± 0.0120**	**0.4436 ± 0.0007**	**0.4510 ± 0.0051**	**0.9896**	0.9880

The indicators including true positive rate(TPR), false positive rate(FPR), precision and recall related to ROC curve and PR curve were calculated as follows:

31 $$TPR=\frac{TP}{TP+FN}$$

32 $$FPR=\frac{FP}{TN+FP}$$

33 $$Precision=\frac{TP}{TN+FP}$$

34 $$Recall=\frac{TP}{TP+FN}$$

In addition, the accuracy is defined as below:

35 $$Accuracy=\frac{TP+TN}{TP+TN+FP+FN}$$

Here, TP and TN represent the number of positive and negative samples predicted correctly, respectively. FN and FP separately denote the number of positive and negative samples that are incorrectly identified.

As shown in Table 2, it is obvious that GSAMDA can achieve the highest AUC values of 0.9496 ± 0.0005 and 0.9308 ± 0.0120 respectively based on both MDAD and aBiofilm, while HMDAKATZ can achieve the second highest AUC values of 0.8712 ± 0.0010 and 0.8993 ± 0.0021separately based on both MDAD and aBiofilm, which are 8.19% and 4.39% lower than that of GSAMDA respectively. Meanwhile, GSAMDA also obtained the highest AUPR values of 0.4436 ± 0.0007 and 0.4510 ± 0.0051 respectively based on both MDAD and aBiofilm. Moreover, the best accuracy of GSAMDA performs better than most compared methods.

### Hyperparameter sensitivity analysis

Considering that there are several hyperparameters in our model GSAMDA including the dimension of node topological representation *l*, the dimension of node attribute representation *k*, and the learning rate *lr*_1_ and *lr*_2_ in GAE and SAE separately, therefore, in this section, we would perform a fivefold CV on the MDAD dataset for 10 times and observe the average AUC value to tune these parameter values.

**Fig. 5:**
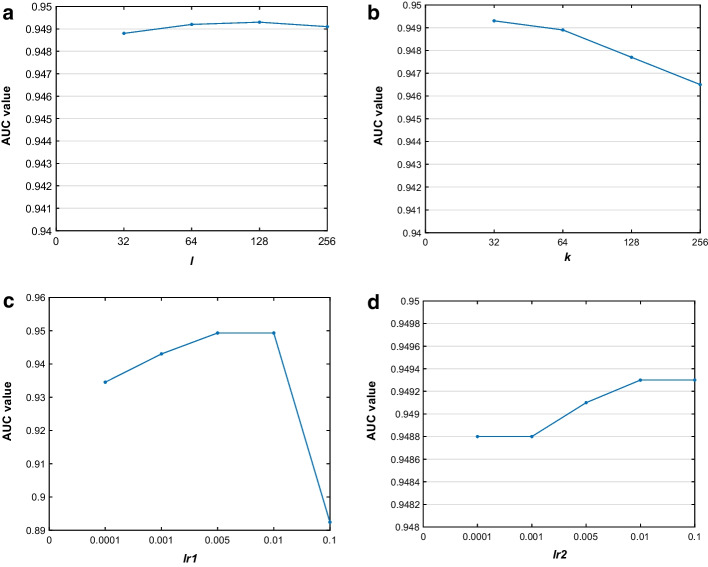
Analysis of the impact of hyperparameters on performance of GSAMDA. The subfigures from (**a**) to (**d**) show the AUC values of the related values of the dimension of node topological representation and node attribute representation, the learning rate of GAE and SAE, respectively

First, we tested the dimension *l* and *k* in the range of {32, 64, 128, 256}, and illustrated the experimental results in Fig. 5a and b, respectively, from which, we found that the dimension has a subtle impact on the performance of GSAMDA. As shown in Fig. 5a and b, when *l* was set to 128 and *k* was set to 32, the performance would be the best. Next, through experimental results, we found that these two parameters for learning rate were important for the performance of GSAMDA, too high or too low of their values would both cause performance degradation of GSAMDA. In experiments, we selected *lr*_1_ and *lr*_2_ in the range of {0.0001, 0.001, 0.005, 0.01, 0.1}, and showed the results in Fig. 5c and d separately, from which, it is easy to see that GSAMDA can obtain the highest AUC values while both *lr*_1_ and *lr*_2_ are set to 0.01.

### Case study

To further validate the performance of GSAMDA, in this section, we would select two popular drugs, Ciprofloxacin and Moxifloxacin, and two microbes, Human immunodeficiency virus type 1 and Mycobacterium tuberculosis, for case studies. During experiments, we selected the top 20 microbes or drugs predicted by GSAMDA based on MDAD for investigation, and then verified that whether these top 20 predicted microbes or drugs have been reported by PubMed literatures.

Ciprofloxacin is a fluorinated quinolone antibiotic with high activity against a wide spectrum of gram-positive and gram-negative bacteria, including methicillin-resistant Staphylococcus aureus, Enterobacteriaceae, and Pseudomonas aeruginosa [35]. For example, *Mycobacterium avium* is highly susceptible to Ciprofloxacin [36]. And it is validated that Ciprofloxacin is an active agent against *Candida albicans* [37]. Besides, the Moxifloxacin [38] is a fluoroquinolone antibiotic, which can treat the social acquired pneumonia caused by Staphylococcus aureus, influenza bacillus, pneumococcus, acute attack of chronic bronchitis, acute sinusitis and so on. Gislason et al. revealed a two-component system that sensitized Burkholderia cenocepacia to moxifloxacin after depletion of the response regulator [39]. Tahoun et al. found that Listeria monocytogenes' antimicrobial susceptibility was most frequently observed for moxifloxacin [40]. Chon et al. demonstrated that most isolates of Clostridium perfringens were susceptible to moxifloxacin [41]. As shown in Tables 3 and 4, among these top 20 predicted ciprofloxacin-associated and moxifloxacin-associated microbes, we found 19 and 17 microbes having been reported by PubMed literatures, which means that the prediction performance of GSAMDA can reach up to 95% and 85%, and demonstrates as well that GSAMDA can achieve satisfactory performance.

**Table 3:** The top 20 predicted Ciprofloxacin-associated microbes

Microbe	Evidence	Microbe	Evidence
Bacillus Subtilis	PMID:33218776	Human Immunodeficiency Virus 1	PMID:9566552
Burkholderia Cenocepacia	PMID:27799222	Klebsiella Pneumoniae	PMID:27257956
Burkholderia Multivorans	PMID:19633000	Listeria Monocytogenes	PMID:28355096
Candida Albicans	PMID:31471074	Mycobacterium Tuberculosis	PMID:30020039
Actinomyces Oris	Unconfirmed	Pseudomonas Aeruginosa	PMID:33875431
Clostridium Perfringens	PMID:29978055	Salmonella Enterica	PMID:6933017
Enteric Bacteria and Other Eubacteria	PMID:27436461	Serratia Marcescens	PMID:23751969
Enterococcus Faecalis	PMID:27790716	Staphylococcus Aureus	PMID:32488138
Escherichia Coli	PMID:26607324	Staphylococcus Epidermidis	PMID:28481197
Haemophilus Influenzae	PMID:27292570	Staphylococcus Epidermis	PMID:10632381

The first column records top 10 microbes, while the third column records top 11–20 microbes

**Table 4:** The top 20 predicted Moxifloxacin-associated microbes

Microbe	Evidence	Microbe	Evidence
Bacillus Subtilis	PMID:30036828	Staphylococcus Aureus	PMID:31689174
Candida Albicans	PMID:21108571	Staphylococcus Epidermidis	PMID:31516359
Clostridium Perfringens	PMID:29486533	Staphylococcus Epidermis	PMID:11249827
Enteric Bacteria and Other Eubacteria	Unconfirmed	Stenotrophomonas Maltophilia	PMID:27257956
Enterococcus faecalis	PMID:31763048	Streptococcus Mutans	PMID:29160117
Escherichia Coli	PMID:31542319	Streptococcus Pneumoniae	PMID:31542319
Haemophilus Influenzae	PMID:11856249	Vibrio Harveyi	Unconfirmed
Listeria Monocytogenes	PMID:28739228	Burkholderia Cenocepacia	Unconfirmed
Pseudomonas Aeruginosa	PMID:31691651	Human Immunodeficiency Virus 1	PMID:18441333
Salmonella Enterica	PMID:22151215	Actinomyces Oris	PMID:26538502

The first column records top 10 microbes, while the third column records top 11–20 microbes

With regards to microbes, mycobacterium tuberculosis is a species of gram-positive, aerobic bacteria that is the etiological agent of tuberculosis which is the leading cause of death from bacterial infections [42]. The bacteria can infect various organs in the human body, causing pulmonary tuberculosis the most common. Moreover, human immunodeficiency virus type 1(HIV-1) is a member of the lentivirus (‘slow-acting’) genus of the family Retroviridae [43]. It is the cause of the Acquired Immunodeficiency Syndrome (AIDS) which is a deadly infectious disease. The top 20 predicted mycobacterium tuberculosis-associated and human immunodeficiency virus type 1-associated drugs are shown in Tables 5 and 6, respectively, from which, we can see that there are 16 and 17 out of top 20 predicted drugs having been validated by PubMed literatures, which further demonstrates the predictive power of GSAMDA as well.

**Table 5:** The top 20 predicted Mycobacterium tuberculosis-associated drugs

Drug	Evidence	Drug	Evidence
Ciprofloxacin	PMID:16270765	Viomycin	PMID:16048924
Epigallocatechin Gallate	PMID:17996734	Capreomycin	PMID:29311078
Tobramycin	PMID:19723387	Ethambutol	PMID:27806932
Curcumin	PMID:24631908	Cloxacillin	PMID:25104892
Vancomycin	PMID:33508482	Aminosalicylic Acid	PMID:26033719
LL-37	PMID:26351280	Amikacin	PMID:29311078
Triclosan	PMID:19130456	3-(pyridin-3-yl)-5-(3-ethoxy-4-hydroxybenzylidene)-2-thioxothiazolidin-4-one	Unconfirmed
Ceftazidime	PMID:28875168	BMAP-28	Unconfirmed
Farnesol	Unconfirmed	3-(4-fluorophenyl)-5-(3-(allyloxy)-4-hydroxybenzylidene)-2-thioxothiazolidin-4-one	Unconfirmed
Pyrazinamide	PMID:26521205	Azithromycin	PMID:7849341

The first column records top 10 drugs, while the third column records top 11–20 drugs

**Table 6:** The top 20 predicted Human immunodeficiency virus type 1-associated drugs

Drug	Evidence	Drug	Evidence
Abacavir	PMID:11797183	Didanosine	PMID:9107385
Amprenavir	PMID:10868554	Efavirenz	PMID:10952598
Atovaquone	PMID:8780816	Emtricitabine	PMID:31879782
Cefditoren	Unconfirmed	Farnesol	Unconfirmed
Cefixime	Unconfirmed	Fosamprenavir	PMID:19515730
Ceftazidime	PMID:11527042	Ganciclovir	PMID:1510405
Cidofovir	PMID:10926733	Indinavir	PMID:8970946
Ciprofloxacin	PMID:9566552	Lamivudine	PMID:12543687
Darunavir	PMID:31879782	LL-37	PMID:17627504
Delavirdine	PMID:9107385	Lopinavir	PMID:20836579

The first column records top 10 drugs, while the third column records top 11–20 drugs

## Discussion and conclusion

Increasing researches have shown that microbes are closely related to human health. Predicting microbe–drug associations can promote microbe-derived therapy and drug discovery. However, traditional wet experiments are time-consuming and expensive and few predictive computational models for microbe–drug associations have been studied. An effective predictive computational model will be a great help for microbe–drug associations discovery.

In this paper, we designed a novel calculation model called GSAMDA based on both GAT and SAE for possible microbe–drug association prediction. In GSAMDA, we first constructed a heterogeneous network based on known microbe–drug associations. And then, the GAT- and SAE-based modules were established to learn the topological representations and the attribute representations of microbe and drug nodes in the heterogeneous network respectively. Finally, through combining the node topological representations and attribute representations with multiple original node features of nodes in the heterogeneous network, the microbe feature matrix and drug feature matrix would be constructed to infer potential microbe–drug associations. Experimental results of both comparison with five state-of-the-art competitive prediction methods and case studies demonstrated the superior performance of GSAMDA and its great potential for drug discovery.

It should be noted that some limitations still exist in GSAMDA. Firstly, the microbe–drug association matrix is sparse and it will affect the performance of the model to some extent. Moreover, not all microbes(drugs) have diseases associated with them, and there are some defects in using microbe(drug)–disease association as attribute feature. Finally, we can incorporate more biological information, such as microbe-microbe interactions and drug–drug interactions, to improve the performance of the model.

## Data Availability

The data and code can be found online at: https://github.com/tyqGitHub/TYQ.
